# Saccadic Movement Strategy in Common Cuttlefish *(Sepia officinalis)*

**DOI:** 10.3389/fphys.2016.00660

**Published:** 2017-01-05

**Authors:** Desiree Helmer, Bart R. H. Geurten, Guido Dehnhardt, Frederike D. Hanke

**Affiliations:** ^1^Sensory and Cognitive Ecology, Institute for Biosciences, University of RostockRostock, Germany; ^2^Department of Cellular Neurobiology, Schwann-Schleiden Research Center, Georg-August-University of GöttingenGöttingen, Germany

**Keywords:** cephalopods, optic flow, vision, motion vision, prototypical movements, saccades

## Abstract

Most moving animals segregate their locomotion trajectories in short burst like rotations and prolonged translations, to enhance distance information from optic flow, as only translational, but not rotational optic flow holds distance information. Underwater, optic flow is a valuable source of information as it is in the terrestrial habitat, however, so far, it has gained only little attention. To extend the knowledge on underwater optic flow perception and use, we filmed the movement pattern of six common cuttlefish *(Sepia officinalis)* with a high speed camera in this study. In the subsequent analysis, the center of mass of the cuttlefish body was manually traced to gain thrust, slip, and yaw of the cuttlefish movements over time. Cuttlefish indeed performed short rotations, saccades, with rotational velocities up to 343°/s. They clearly separated rotations from translations in line with the saccadic movement strategy documented for animals inhabiting the terrestrial habitat as well as for the semiaquatic harbor seals before. However, this separation only occurred during fin motion. In contrast, during jet propelled swimming, the separation between rotational and translational movements and thus probably distance estimation on the basis of the optic flow field is abolished in favor of high movement velocities. In conclusion, this study provides first evidence that an aquatic invertebrate, the cuttlefish, adopts a saccadic movement strategy depending on the behavioral context that could enhance the information gained from optic flow.

## Introduction

It is largely unknown which cues underwater species use to navigate safely through their environment. Only recently optic flow, defined as the visual pattern elicited on the retina of a moving observer (Gibson, 1950), has reattracted notice as possible source of information in the underwater world (Gläser et al., 2014; Scholtyssek et al., 2014). Extending these studies, Geurten et al. (under revision) showed that harbor seals adopt a saccadic movement strategy comparable to terrestrial species such as insects (see e.g., Collett and Land, 1975; Zeil, 1986, 1996; Zeil et al., 1996; Van Hateren and Schilstra, 1999; Tammero and Dickinson, 2002; Ribak et al., 2009; Boeddeker et al., 2010; Geurten et al., 2010; Kress and Egelhaaf, 2012, 2014) or birds (Eckmeier et al., 2008; Kress et al., 2015; Pete et al., 2015). These animals perform short rotations of the eyes, the head, or the body depending on species. These rotations are called saccades and minimize the time during which spatial information cannot be derived from the optic flow field as all objects irrespective of their distance to the observer move with the same rotation velocities (Koenderink and van Doorn, 1987). In contrast, these animals predominantly translate through their environment as translational movements allow the extraction of distance information from optic flow as the closer the objects, the faster they move.

To analyze if the saccadic movement strategy is as widespread underwater as it is in the aerial habitat, we studied the movement pattern of another aquatic animal, the common cuttlefish *(Sepia officinalis)*, which has a completely different movement pattern and lifestyle than seals. Furthermore, their last common ancestors are the bilaterians, which lived ≈500 Mio years ago. Cuttlefish are benthic cephalopods which have well-developed eyes and good vision (Budelmann, 1995; Hanlon and Messenger, 1996). Their eyes are very mobile and show optokinetic responses as a response to moving stimuli (Collewijn, 1970; Messenger, 1970), and eye movements seem to precede and compensate body movements during rotations (Messenger, 1968; Collewijn, 1970).

Cuttlefish actively prey upon fish or crustaceans which they capture by ejecting their extensible tentacles or by jumping on and enveloping the item with all arms, called arm attack (Sanders and Young, 1940; Wilson, 1946; Messenger, 1968; Nixon and Dilly, 1977; Duval et al., 1984). The latter occurs mainly with slow moving prey. Their attacks on prey are predominantly visually-driven with an attention, positioning, and seizure phase (Sanders and Young, 1940; Messenger, 1968, 1977; Chichery and Chichery, 1988). During an attack, cuttlefish seem to estimate the distance to the prey item as (1) they either retreat from or approach the object, (2) they modify the ocular convergence depending on the distance to the prey object (Messenger, 1968), (3) unilaterally blinded animals or animals in which the optic commissure and the basal lobes are divided are less accurate in seizing prey in comparison to normal sighted animals (Messenger, 1977), and (4) they seem to possess size constancy (Messenger, 1977). Cuttlefish might gain distance and depth information by accommodation as a change in refractive state was observed just before the cuttlefish attacked the prey item (Schaeffel et al., 1999), by the W-shaped pupil being a monocular in-or-out-of-focus detector (Schaeffel et al., 1999; Mäthger et al., 2013) or by using texture density gradients (Josef et al., 2014). Another mechanism that would allow for visual distance estimation in a feeding and non-feeding context, as outlined above, is translational optic flow. As a first approach to analyze if optic flow perception is used in cuttlefish to measure distances, we recorded the movement pattern of a small group of six cuttlefish to analyze if cuttlefish move their bodies saccadically in line with the saccadic movement strategy documented for other animals.

## Materials and methods

### Experimental animals

The experiment was conducted with six cuttlefish *(S. officinalis)* individuals at the Marine Science Center, Rostock, Germany. The cuttlefish hatched in captivity in January 2015 at the Max-Planck-Institute for Brain Research, Frankfurt, Germany, and were thus half a year old when their movement pattern was recorded. The animals were kept in accordance with current maintenance protocols for cephalopods (Andrews et al., 2013; Smith et al., 2013; Fiorito et al., 2014, 2015) in line with the Directive 2010/63/EU. Approval (6712GH00113) was given by local authorities (Staatliches Amt für Umwelt und Natur Rostock) according to §42 of the German law on nature protection.

One up to two cuttlefish individuals shared one compartment of a 3000 l sea water aquarium system. Water quality was regularly controlled, and salinity and temperature were adjusted to 32 g/kg and 21°C, respectively. The bottom of the aquarium was covered with small pieces of corals or sand, which allowed the cuttlefish to burry themselves. The tank was artificially illuminated (daylight spectrum) with a natural day–night-cycle of 12 h/12 h. The day cycle included a phase of dawn and dusk of 1 h. To ensure a balanced diet, the animals were fed one to three times a day with *Palaemon* sp., deep frozen fish or fish pieces from *Osmerus eperlanus, Sprattus sprattus*, or *Clupea harengus* or shrimp *(Pandalus borealis)*.

### Experimental procedure

For 4 days, during which the movement pattern of cuttlefish was recorded, cuttlefish were housed together in a large compartment (150 × 51.5 × 85 cm) in a group of six individuals to maximize the time at least one individual was visible in the field of view of the camera. Within the field of view of the camera, a red PVC board (50 × 50 cm) was placed on the bottom of the compartment. The cuttlefish were lured onto the board with *Palaemon* sp. that were inserted in fasteners. The fasteners could be moved with fine thread not causing water disturbances at the water surface that would have lowered the quality of the recordings. The cuttlefish attacked the lure and removed the prey from the nut. Filming cuttlefish on the red board increased the contrast of the otherwise cryptically colored animals, which facilitated video analysis. To additionally facilitate video analysis, the luminance of the region of interest was increased with external lamps that were switched on only during filming.

The movement pattern of the cuttlefish was filmed with a black-and-white high speed camera (Photon focus DR1-D1312-200-G2, Lachen, Switzerland) with an objective with a focal length of 16–100 mm (Varifocal SC-VZ-16100M, SpaceCom, Tokyo, Japan) at 200 frames/s. The camera was installed 50 cm above and orthogonal to the water surface. We are confident that we can adequately describe the movements of the cuttlefish from video recordings from above as we moved the prey items mainly close to the bottom avoiding large vertical movements and as the movement of cuttlefish with their benthic lifestyle (Russell-Hunter, 1979) is predominantly two-dimensional. This assumption is supported by only small vertical movements amounting to 5.7 ± 4.7% quantified on the basis of the maximal difference in dorsal mantle length of the cuttlefish.

### Video analysis

**Figure 1:**
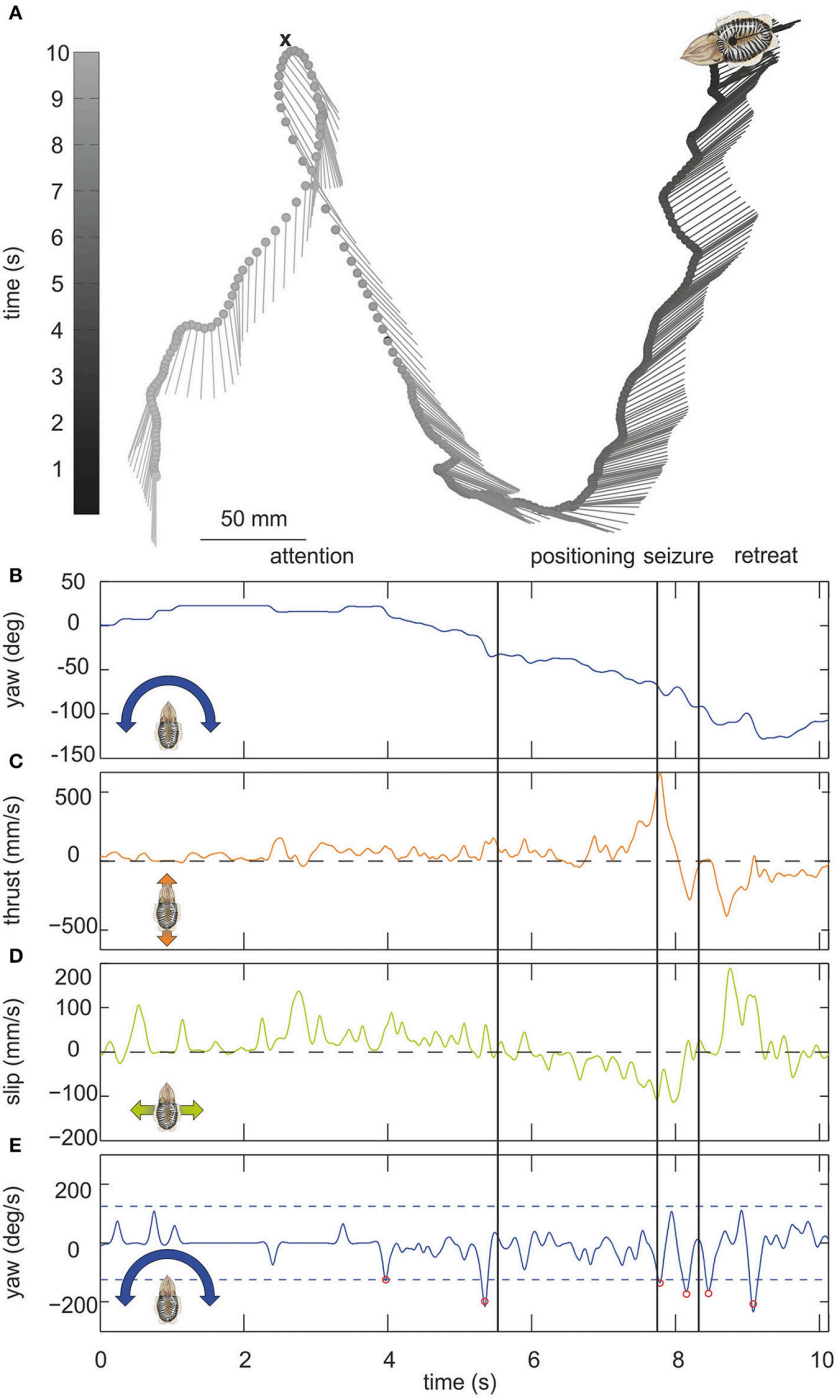
**(A)** Example trajectory of a moving cuttlefish. The cuttlefish moved in forward direction from the lower left corner to the upper tip of the loop where it captured a small crab (indicated by a cross). It then moved backwards up to the upper right corner. The lines mark the long axis of the cuttlefish, the dots indicate the center of mass of the cuttlefish body over time (in s) which is depicted in gray scale from light gray representing the start of the movement to dark gray end of the movement. The position of the center of mass is plotted every 100 ms. The scale for dimensions is 50 mm. **(B–E)** Parameters of the cuttlefish's movement with **(B)** the yaw angle (in °), **(C)** the thrust and **(D)** slip velocities (mm/s), and **(E)** yaw velocity (in °/s). In **(E)** saccades, defined by velocities ≥125°/s (dashed lines), are marked by red circles. Vertical lines mark the end/start of the phases attention, positioning, seizure and retreat as indicated above the figures.

The video recordings were analyzed with the help of the software ivTrace Image Analysis (https://opensource.cit-ec.de/projects/ivtools). We analyzed all video sequences obtained and only omitted those video recordings with obvious interactions between cuttlefish individuals. On the recordings, the center of mass of the cuttlefish body was tracked over time. Additionally, the orientation of the cuttlefish body and its coordinates in a two-dimensional space were determined. Using these parameters, the movement of the cuttlefish could be described as thrust, slip, and yaw movement defined as for-/backward movement, movement to the side, and rotations around the body axis (Figure 1 insets). Velocities of these three movement directions were calculated from the change in position and orientation between subsequent frames. Movements with velocities exceeding 3000°/s or 7000 mm/s were classified as artifacts and were consequently excluded from the analysis.

The subsequent analysis steps were conducted with the help of custom written programs in Matlab (The Mathworks, Natick, Massachusetts, USA). The velocity of yaw, thrust, and slip movements of the body were determined by calculating the angle covered or the distance moved by the body between two frames. To convert distances moved from pixel into mm, the size of the red board, which was placed on the bottom of the tank, was taken as scale.

Furthermore, a cluster analysis was conducted to describe the prototypical movement pattern of cuttlefish. Therefore, the velocity data was z-scored (normalized to a 0 mean and a standard deviation of 1) to account for numeric differences between rotational and translational speeds. For every frame on which the animal had moved a three-dimensional velocity vector consisting of thrust, slip, and yaw velocity was then fed into a hierarchical agglomerative clustering routine (MatLab Statistics Toolbox). As the whole data set was too large to be clustered at once, it was split up into 2% chunks that were clustered sequentially (Hastie et al., 2009a; Murtagh and Contreras, 2012). We used the squared Euclidean distance and “Ward criterion” to build hierarchical clusters. This first step of analysis rendered a possible number of clusters between 2 and 50. We subsequently clustered the complete data set again with the k-means algorithm (MacQueen, 1967; Milligan and Cooper, 1987; Hastie et al., 2009b). We clustered all classes between 2 and 20. For 20–50 classes, only every fifth class was analyzed because we rarely saw stable cluster combinations with these large numbers of classes (Geurten et al., 2010, 2014; Hofmann et al., 2014). To determine the number of classes that represent our data best, we used the quality and stability criteria described in Braun et al. (2010).

### Statistical analysis

We employed Fisher's permutation tests (Fisher, 1954) on the differences between the medians of different experimental groups, which were refined by various authors (see e.g., Crowley, 1992; Ernst, 2004). We corrected the *p*-values with the Benjamini–Hochberg false detection rate procedure (Benjamini and Hochberg, 1995; Groppe et al., 2011) using the Matlab implementation of Benjamini and Hochberg's procedure by David M. Groppe (https://de.mathworks.com/matlabcentral/fileexchange/27418-fdr-bh).

## Results

Altogether 202 videos including 256,830 single frames could be analyzed. Figure 1A illustrates a characteristic trajectory of a cuttlefish moving over a time frame of ≈10 s. The black line connecting the dots describes the movement of the center of mass of the body over time, whereas the short lines represent the yaw orientation of the body. During the first phase of the movement, the cuttlefish was moving forward positioning itself with the moving prey item. This phase ends when the cuttlefish jumped on the prey item at the upper tip of the loop. The seizure of the prey was accompanied by fast thrust and slip movements (Figures 1C,D). In the last phase, it retreated from the point of prey capture with a fast back- and sideward movement (Figures 1C,D).

**Figure 2:**
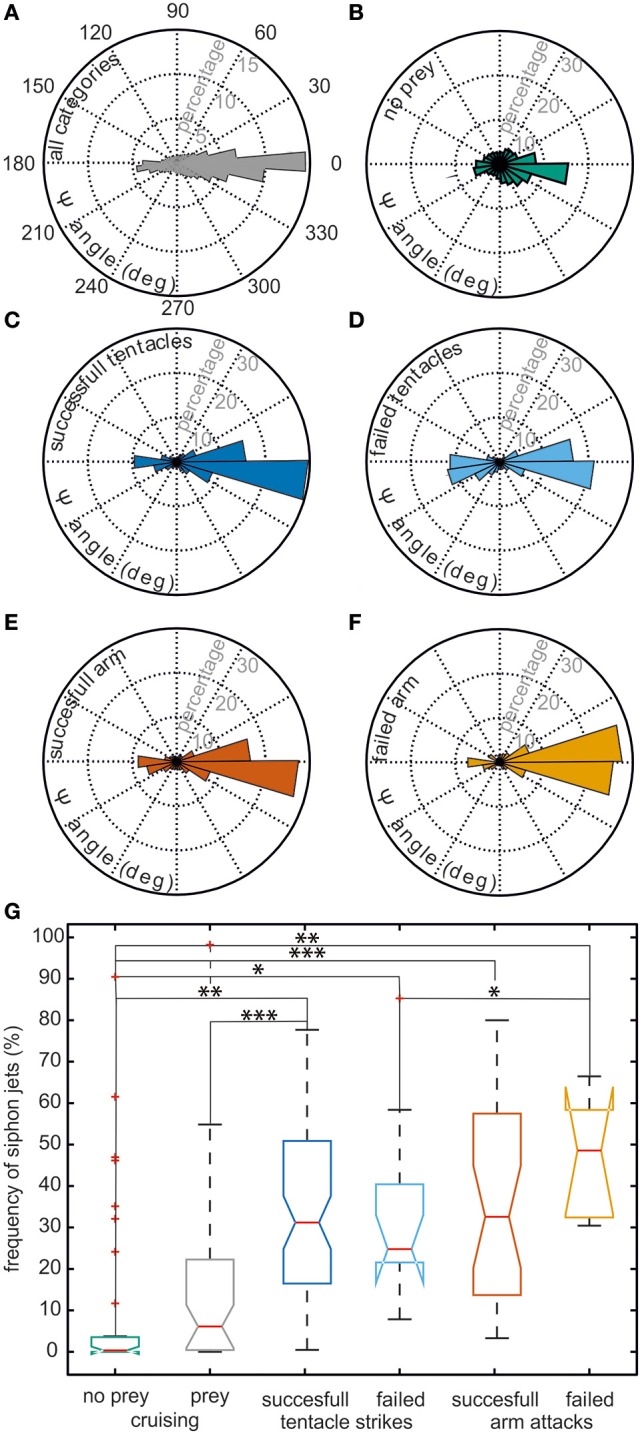
**Context dependent analysis of the ψ-angle distribution and usage of siphon jet propulsion**. Cuttlefish trajectories were categorized according to the following contexts: successful tentacle strikes (*n* = 71 trajectories), failed tentacle strikes (*n* = 13), successful arm attacks (*n* = 31), failed arm attacks (*n* = 7), cruising sequence with prey in the animals' vicinity (*n* = 43), and cruising sequences without prey (*n* = 37). **(A–F)** The angle between the body long axis and the movement direction (ψ-angle) is plotted as a rose plot. A ψ-angle of 0° codes for a forward movement, whereas a ψ-angle of 180° describes a backward movement. The ψ-angle distribution occurring in all trajectories is depicted in **(A)**, Panels **(B–F)** show the ψ-angle distribution for different contexts. ψ-angle obtained from trajectories in which **(B)** no prey animals were present, **(C)** successful or **(D)** failed tentacle strikes, or **(E)** successful or **(F)** failed arm attacks were documented. In **(G)**, the frequency with which siphon jet propulsion occurred during different behavioral contexts is plotted. There is no significant difference when comparing different types of attack and their outcome. However, the frequency of jet propulsion differs significantly between cruising and attacks and between the presence of prey items or their absence. Significance was determined using Fisher's exact permutation test and corrected via Benjamini–Hochberg false detection rate procedure (see Section Statistical Analysis). ^*^p < 0.05, ^**^p < 0.01, and ^***^p < 0.001.

It is evident from this example trajectory that the body was not necessarily aligned with the swimming direction. This phenomenon was also generally revealed by the ψ-angle analysis (Figures 2A,B) that describes the angle between the body long axis and the movement direction. Only during a phase at the beginning of the movement and in a short retreat phase after prey capture of the example trajectory (Figure 1), a clear alignment of body and the direction of movement could be observed. In general, during hunting trajectories, there was a clear bias to ψ-angle of either 0° or 180° (Figures 2C–F). This emerged from the cuttlefish's preference to align prey and body axis during the phases of the attack (Messenger, 1968) and moreover to use its fast siphon jet propulsion to approach prey and to leave the place where it has just caught its prey on the fastest way. Siphon propulsion was used significantly more often during attacks than during normal cruising (*p* < 0.001 Fisher's permutation test; Benjamini Hochberg false detection rate correction; Figure 2G). A pronounced biphasic distribution of the ψ-angle was especially prominent during failed attempts to catch a prey item. After an unsuccessful tentacle strike, the animal moved backward to aim for its target a second time (Figure 2D). In contrast, the 180° ψ-angle component is largely missing if the cuttlefish has unsuccessfully tried to seize the prey with an arm attack as they did not retreat in this situation but continued to follow the prey item (Figure 2F).

**Figure 3:**
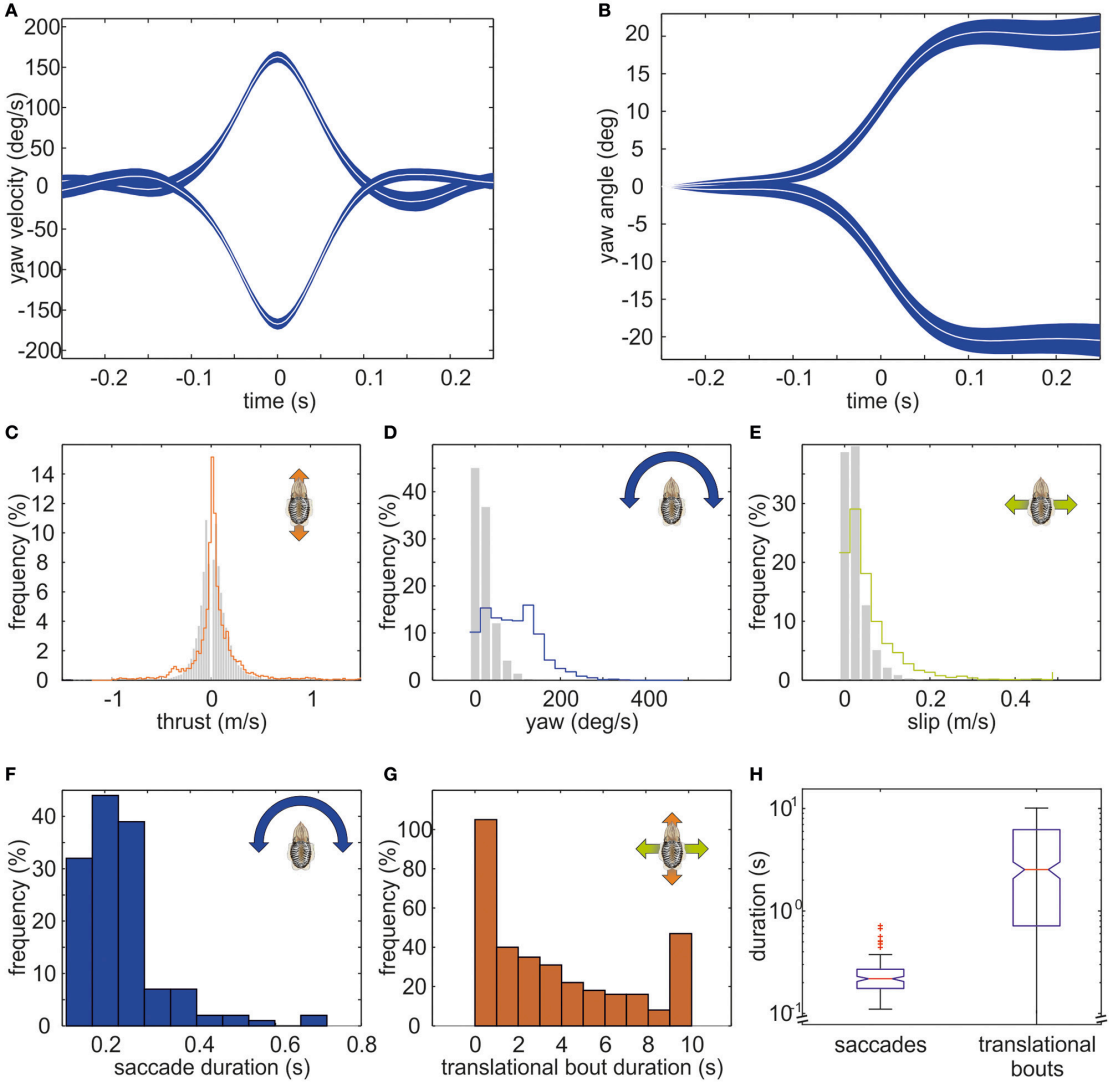
**Detailed analysis of saccades of cuttlefish**. The mean course of saccades in cuttlefish (*N* = 136) is depicted as mean yaw velocity (in °/s) in **(A)** and as mean yaw angle (in °) in **(B)**. White curves indicate the median. The blue shaded area depicts the 95% confidence interval of the median. Saccades to the right (positive values) and to the left (negative values) are displayed separately. In general, saccades are short events that last for 217.5 ms on average. During a saccade, the body reached a mean rotation velocity of 152°/s (±*SD*) and covered a mean rotation angle of 18.8°. **(C**–**E)** Frequency (in %) of translations (gray bars) and saccades (colored lines) during **(C)** thrust, **(D)** yaw, and **(E)** slip movements. While the distribution of thrust velocities is rather similar during translations and saccades, sideways and yaw velocities are faster during saccades. **(F)** Frequency (in %) with which saccades of different durations occurred (binned in 0.1s). **(G)** Frequency (in %) with which translational bouts of a specific duration (in s) occurred. **(H)** Boxplots for the duration (in s; logarithmic scale) of saccades and translational bouts with the boxes indicating the quartiles, the red line indicating the median, and the 1.5 interquartile distance is shown by the whiskers. Outlying data points are marked with red crosses. The notches in the boxes exhibit the 95% confidence interval of the median. Saccades are significantly longer events than translations (*p* < 0.01).

The example trajectory moreover shows that there are periods during which the body showed a constant orientation over time (Figure 1B). However, changes in orientation were fast and short, which is characteristic for saccadic turns (Figures 1B,E). During this example movement, six saccades marked by red circles in Figure 1E could be detected. Saccades were generally defined as short rotations reaching velocities of ≥125°/s. Figure 3 characterizes all 136 saccades documented in the video material. During these saccades, the body reaches a mean rotation velocity of 168 ± 44.6°/s (Figure 3A). Generally, saccades vary in velocity between 125 and 343°/s, and the body rotates with a mean yawing angle of 20.6 ± 16.2 ms (Figure 3B). The angles covered by the body from frame to frame ranged between 9 and 85°. Thrust velocity is on average faster during saccades than during translational bouts (154–124 mm/s; Figure 3C), as are slip and yaw velocities increased (slip: 28–66 mm/s, yaw: 24–88°/s; Figures 3D,E). This shows that translational and rotational velocities do not coincide, but that fast rotations are segregated from other movements in line with a saccadic movement strategy. Cuttlefish saccades range in duration from 110 to 720 ms with a mean duration of 237 ± 98 ms (Figure 3F). In contrast, cuttlefish perform translations lasting 3.7 ± 3.5s ms on average (Figure 3G). Thus, translational bouts are significantly (*N* = 202, *p* < 0.001, Fischer's exact permutations test) longer than saccades (Figure 3H).

**Figure 4:**
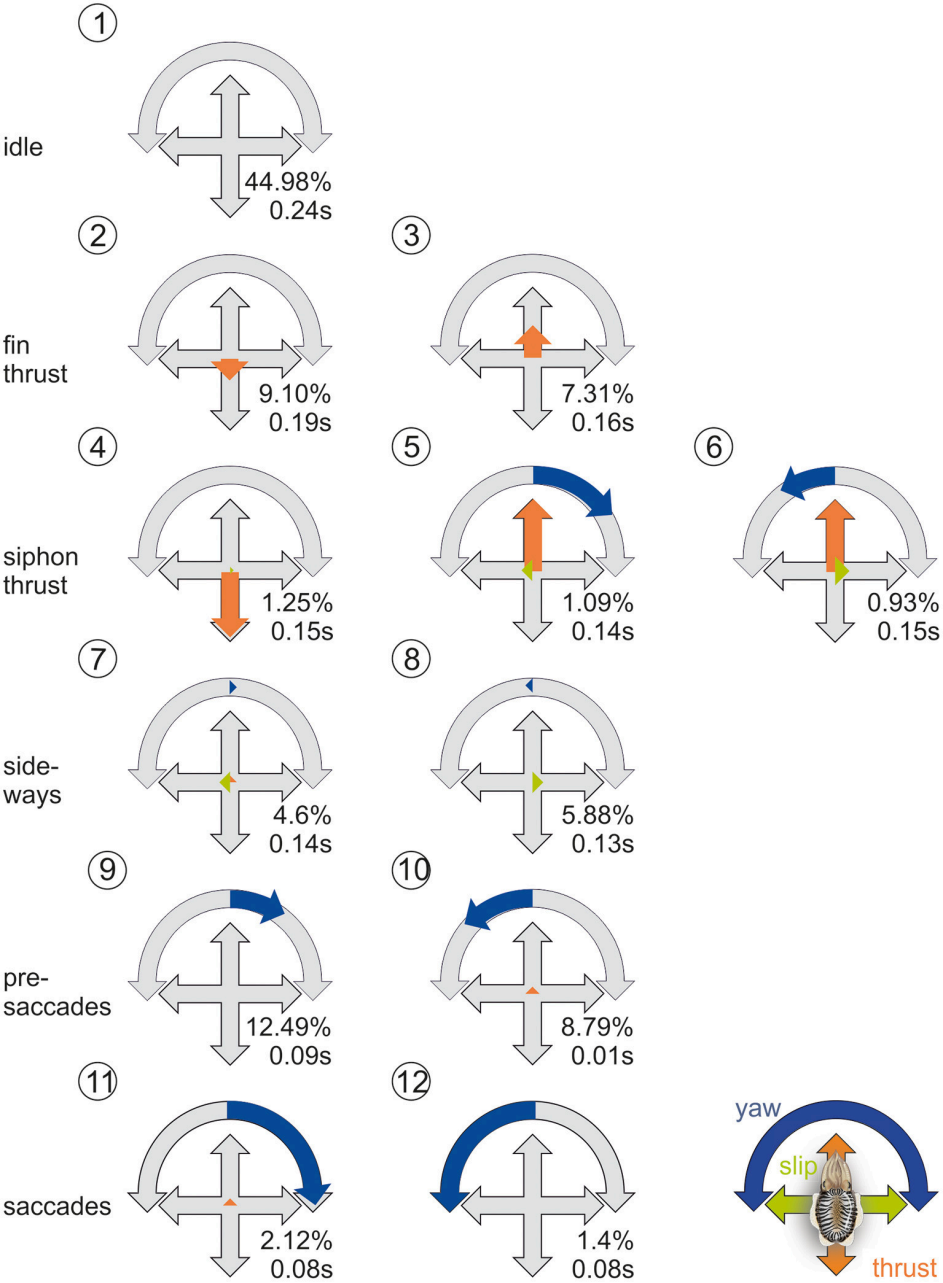
**Prototypical movements of the common cuttlefish**. Normalized thrust, slip and yaw rotation velocity (thrust and slip were normalized to their maximum, yaw on its absolute maximum) for the 12 clusters as well as frequency as percentage of total events (*N* = 256,628) and mean duration of the behavioral element. PM2-6 are thrust dominated, PM7-8 describe slight sideways movements, and PM9-12 are characterized by an increase of rotational yaw movements. Blue arrows denote yaw rotations to the left or right, green arrows denote slip movements to the left or right, orange arrows denote thrust movements to the front or back.

The cluster analysis yielded the best stability and quality for 12 clusters. In those clusters (Figure 4), the two movement types of cuttlefish (Russell and Steven, 1930) are apparent: the first type of movements is elicited by a complex movement of the fins with an average movement velocity of 138 m/s, the second by the jet expelled from the siphon during which the cuttlefish reached velocities of 430 m/s, which they predominantly use during hunting (Figure 2G). These movement types go along with two different strategies. Whereas, during fin motion, rotations and translations are clearly separated (cluster 2, 3, 11, 12, Figure 4), rotations and translations are coupled during jet propulsion (cluster 4–6, Figure 4). Overall forward movements, however, dominate over back- and sideward movements as also revealed by the ψ-angle analysis (Figures 2A–F).

## Discussion

The results of this study revealed that cuttlefish employ a saccadic movement strategy. We analyzed body movements as a first approach as eye movements could not be resolved on our recordings. However, we assume that the eyes of cuttlefish also move saccadically in support of the saccadic body movements. Evidence supporting this hypothesis stems from previous studies (Messenger, 1968; Collewijn, 1970; Chichery and Chichery, 1987, 1988) in which it was shown that cuttlefish perform eye movements, ocular saccades in particular, in compensation of body rotations. During the saccades, only the rotation direction and velocity is perceptible from the optic flow field. This information could be useful for the animal's positioning as it is directly available in contrast to information from statocysts (Budelmann et al., 1973; Budelmann, 1979), which have a longer latency. If and how the optic flow information is integrated into the signal of the statocysts has to be analyzed in future studies.

We did not observe directed movements of the head in relation to the mantel cavity, similar to the head stabilization of birds (Pratt, 1982; Wohlschläger et al., 1993). Although, a closer investigation of the mantel orientation might reveal further stabilization strategies, the most obvious place for further gaze stabilization would be the moveable eyes of *S. officinalis*. In conclusion, by performing body saccades most likely in combination with eye movements, cuttlefish reduce the time of rotations as rotations complicate the extraction of distance information from optic flow. Thus, this study most likely adds a mechanism to the already reported distance/depth estimation mechanisms in cuttlefish (Schaeffel et al., 1999; Mäthger et al., 2013; Josef et al., 2014). Distance estimation from optic flow offers the advantage that it provides distance information for much larger distances than the alternative mechanisms. Moreover these data add to the overall picture that all moving animals irrespective of their eye type, mode of locomotion, visual environment including the medium, in which they operate, use optic flow to guide their movements.

In contrast to terrestrial species as well as to harbor seals, cuttlefish show a context dependent strategy as revealed by the cluster analysis. During fin motion, cuttlefish move at relatively low speeds and clearly separate their body movements into saccades and translations. This behavior corresponds to the saccade movement strategy documented in terrestrial species (see e.g., Collett and Land, 1975; Schilstra and Hateren, 1999; Blaj and van Hateren, 2004; Eckmeier et al., 2008; Ribak et al., 2009; Geurten et al., 2010, 2014; Kress and Egelhaaf, 2012) as well as in the harbor seal (Geurten et al., under revision). In contrast, the cuttlefish abolishes optic flow analysis to gain distance information when it moves its body at high velocity with the pulsed jet of its siphon. Thus, cuttlefish seem to trade their swimming velocity and the extraction of distance information form optic flow depending on the context. Siphon movements predominantly occurred shortly before and after a prey capture event. In this situation, the cuttlefish seem to primarily focus on speed to catch the prey item and to leave the location of prey capture. Especially under competition pressure, the best strategy for an animal is to escape in a straight line with high velocities. Such an escape behavior has e.g., also been shown for the African ball-rolling dung beetle that rolls its dung ball on a straight path from the dung pile at which it encounters intense competition among conspecifics (Byrne et al., 2003; Dacke et al., 2003a,b,c, 2011, 2013). A very fast escape movement in cuttlefish might have evolved because they are soft-bodied animals with many predators.

This study provided a detailed characterization of body saccades in cuttlefish. Cuttlefish saccades were defined as rotations exceeding a rotation velocity of 125°/s. This velocity threshold seems conservative when compared to the results of optokinetic studies (Collewijn, 1970; Messenger, 1970). In these studies, low gain optokinetic responses up to a rotational velocity of the optokinetic drum of only 35°/s were reported. However, the gain function published by Collewijn (1970) suggests that the cuttlefish might have also responded to higher rotational velocities if these had been tested. This claim is supported by Boulet (1960) who documented ocular reactions to a target movement of up to 51°/s and also by Cartron et al. (2013) who state that cuttlefish followed drum movements up to 100°/s but failed at a stimulus velocity of 130°/s. Cuttlefish body saccades lasted for 217.5 ms on average. It is very probable that the eyes even move faster although Collewijn (1970) reported that it took a cuttlefish eye 0.5 s to complete a saccade. The cuttlefish rotated their bodies by an angle of 9–85°. From observations and as documented by Messenger (1968), cuttlefish rotate their eyes together with the body by almost 180° in the first phase, the attention phase of their attack. The overall goal in this phase of the attack is to align the optical and the prey axis. It is very likely that we did not record such wide angles as we inserted the prey predominantly within the anterior visual field of a cuttlefish close to the platform. Thus, there was no need for the cuttlefish to turn by a large angle. The behavior we documented thus predominantely describe the movement pattern of cuttlefish in the second and third phase of the attack, positioning, and seizure (Messenger, 1968).

Cuttlefish and seal body saccades (Geurten et al., under revision), the only saccades documented for swimming animals up to now, are very similar in respect to their mean and maximum rotation velocities. These velocities are achieved by slightly smaller angles covered in a shorter period of time in cuttlefish in comparison to harbor seals that rotate in larger angles which also takes more time. These differences are most likely due to the larger body size of harbor seals as compared to cuttlefish. Body saccades of these two aquatic species are surpassed in rotation velocity by most flying species (Blaj and van Hateren, 2004; Eckmeier et al., 2008; Geurten et al., 2010), which is probably due to the higher viscosity and density of water vs. air. Whereas, harbor seals change between active swimming and gliding (Geurten et al., under revision), cuttlefish switch between two active swimming modes, fin motion, and jet propelled swimming. However, the movements made by cuttlefish are characterized by movements along as well as perpendicular to the body axis, the latter not occurring in harbor seals.

In conclusion, this study revealed that cuttlefish move their bodies saccadically thereby probably optimizing the extraction of distance information from optic flow. Future studies however need to be performed to proof the usage of optic flow in *S. officinalis*. Cuttlefish change between a saccadic moment strategy and high movement velocities, during which they abolish the separation of rotational and translational movements, a flexibility that is unique till now. Moreover the finding of a saccadic movement strategy in another aquatic species besides harbor seals suggests that this strategy might be as wide-spread underwater as it is in the terrestrial habitat.

## Author contributions

All authors designed the study, DH, BG, FH collected and analyzed the data, BG, FH wrote the manuscript, all authors edited and approved the manuscript.

## Funding

This study was supported by a grant of the VolkswagenStiftung to GD.

### Conflict of interest statement

The authors declare that the research was conducted in the absence of any commercial or financial relationships that could be construed as a potential conflict of interest.
